# Cancer Informatics 2025: Bringing foundation models and multimodal cancer data to the forefront

**DOI:** 10.1055/s-0046-1816608

**Published:** 2026-11-03

**Authors:** Sanjay Aneja, Ravi B. Parikh

**Affiliations:** 1 Yale Cancer CenterNew HavenCTUSA; 2 Department of Therapeutic Radiology, Yale School of MedicineNew HavenCTUSA; 3 Department of Bioinformatics and Data Science, Yale School of MedicineNew HavenCTUSA; 4 Emory UniversityAtlantaGAUSA; 5 Winship Cancer InstituteAtlantaGAUSA

**Keywords:** Neoplasms, informatics, health information technology, clinical implementation, foundation models, multimodal AI, artificial intelligence

## Abstract

**Objectives:**

To summarize significant research contributions on cancer informatics published in 2024, an extensive search using PubMed/MEDLINE was conducted to identify the scientific contributions published in 2024 that address topics in cancer informatics. The selection process was composed of three steps: 1) 15 candidate best papers were first selected by the two section editors; 2) external reviewers from internationally renowned research teams reviewed each candidate best paper; and 3) the final selection of two best papers was conducted by the editorial board of the IMIA Yearbook.

**Results:**

The two selected best papers demonstrate advances in the clinical implementation of cancer informatics methodologies. Both studies highlight the potential for multimodal data and foundation models as well as prospective implementation of AI to influence cancer care.

**Conclusions:**

Cancer informatics has evolved into a sophisticated discipline within bioinformatics, with increasing emphasis on multimodal artificial intelligence and foundation models that integrate diverse data types for comprehensive cancer analysis. As these innovative approaches mature, the cancer informatics field prioritizes robust prospective validation and deployment strategies to ensure these technologies deliver meaningful improvements in patient care and clinical outcomes.

## 1. Introduction

Cancer informatics (CI) encompasses a multidisciplinary domain centered on three core objectives: i) structuring heterogeneous data into clinically actionable formats for healthcare providers, researchers, and patients [ 1 , 2 ]; ii) leveraging computational approaches to enhance cancer therapeutics and patient management [ 3 ]; and iii) extracting novel biological and clinical insights from complex datasets [ 4 ]. This year’s Cancer Informatics section emphasizes the critical transition toward clinical translation and implementation. Foundation models—sophisticated artificial intelligence (AI) architectures trained on massive, diverse datasets to learn generalizable representations—have emerged as powerful tools for cancer analysis. Multimodal AI systems, which integrate multiple data modalities such as medical imaging, clinical records, genomic profiles, and pathology slides, offer unprecedented opportunities for comprehensive cancer assessment. While historical cancer informatics research concentrated primarily on algorithmic development and proof-of-concept studies, contemporary efforts prioritize real-world deployment and clinical validation. The selected papers of 2024 exemplify this paradigm shift, featuring informatics solutions that demonstrate either rigorous prospective evaluation through randomized controlled trials or practical scalability across diverse healthcare environments.

## 2. Paper Selection Method

One electronic database was searched—PubMed/MEDLINE. The search was performed in January 2025 to identify peer-reviewed journal articles related to cancer informatics research written in the English language and published in 2024. The following search was implemented:

**Table:** 

SEARCH QUERY
((“Neoplasms”[Mesh] OR “chemotherapy”) AND (“Informatics”[Mesh] OR “cancer informatics” OR “ontologies” OR “machine learning” OR “artificial intelligence” OR “multimodal” OR “large language model” OR “foundation model”) AND (hasabstract[text] AND (“2024/01/01”[PDAT] : “2024/12/31”[PDAT]) AND English[lang])) NOT (“Radiotherapy Planning, Computer-Assisted”[Mesh]) NOT (“Radiotherapy, Computer-Assisted”[Mesh])

This is identical to the search for papers in 2024, except that we also added terms related to AI and machine learning (ML). This search yielded 7,611 results. Next, we excluded review articles, resulting in ~2000 articles for first-pass review. The titles of these articles were blindly screened for relevance, resulting in 109 articles that were reviewed in further depth. The abstract of each of the 109 was blindly reviewed and assigned as potential candidate (n=27) and non-candidate (n=82). Given that the number of potential candidates exceeded ten, these articles were further evaluated to select 15 final candidates.

In accordance with the IMIA Yearbook selection process, the 15 candidate best papers were evaluated by the two section editors, senior editors, and by additional external reviewers (at least four reviewers per paper). [ 5 ] Two papers were finally selected as best papers ( Table 1 ). A content summary of the selected best papers can be found in the appendix of this synopsis.

**Table 1:** Selection of best paper for the 2025 IMIA Yearbook of Medical Informatics for the section Cancer Informatics.

Section Cancer Informatics
• Cui H, Zhao Y, Xiong S, Feng Y, Li P, Lv Y, et al. Diagnosing Solid Lesions in the Pancreas With Multimodal Artificial Intelligence: A Randomized Crossover Trial. JAMA Netw Open. 2024 Jul 1;7(7):e2422454. https://doi.org/10.1001/jamanetworkopen.2024.22454 . PMID: 39028670; PMCID: PMC11259905. • Wang X, Zhao J, Marostica E, Yuan W, Jin J, Zhang J, et al. A pathology foundation model for cancer diagnosis and prognosis prediction. Nature. 2024 Oct;634(8035):970-978. https://doi.org/10.1038/s41586-024-07894-z . Epub 2024 Sep 4. PMID: 39232164.

## 3. Outlook

The 15 candidate papers from 2024 showcase the breadth of contemporary cancer informatics research, spanning foundation model architectures to prospective clinical trials. These investigations encompassed diverse data sources including administrative databases, structured electronic health records, medical imaging modalities, and high-resolution pathology specimens. The two selected exemplars address distinct yet complementary aspects of cancer care: diagnostic enhancement for pancreatic malignancies and comprehensive pathology-based cancer assessment and prognosis. This methodological and clinical diversity underscores cancer informatics’ maturation as a distinct field and demonstrates the expanding potential for data-driven personalized oncology interventions.

Cui *et al.* [ 6 ] conduct a randomized crossover trial to evaluate whether a multimodal AI model combining endoscopic ultrasonographic images and clinical data could improve diagnosis of solid pancreatic lesions compared to conventional approaches. In a study spanning four centers with 628 retrospective patients for model development and 130 prospective patients for validation, 12 endoscopists of varying expertise levels participated in the crossover trial. The authors found that AI assistance significantly improved novice endoscopists’ diagnostic accuracy from 0.69 to 0.90 and sensitivity from 0.61 to 0.91, while interpretability features using Gradient-weighted Class Activation Mapping (Grad-CAM) and SHapley Additive exPlanation (SHAP) analyses reduced rejection rates among experienced clinicians. The multimodal AI model demonstrated robust performance across internal and external datasets (area under the receiver operating curve [AUC] 0.924-0.996) and showed potential to complement existing diagnostic techniques for pancreatic lesion evaluation.

Wang *et al.* [ 7 ] developed the Clinical Histopathology Imaging Evaluation Foundation (CHIEF) model, a multimodal foundation model that integrates histopathology images and clinical data for comprehensive cancer evaluation. The CHIEF model was trained on 60,530 whole-slide images from 19 anatomical sites using complementary unsupervised and weakly supervised pre-training approaches. The model was validated across 19,491 whole-slide images from 32 independent datasets spanning 24 hospitals internationally, demonstrating superior performance in cancer detection, tumor origin identification, genomic profiling, and survival prediction tasks. CHIEF consistently outperformed state-of-the-art methods by up to 36.1%, achieving macro-average AUCs of 0.9397 in cancer detection and successfully addressing domain shift challenges that limit generalizability of existing AI pathology models. The foundation model provides interpretability through attention visualization and represents a significant advancement toward clinical translation of AI in digital pathology by offering robust performance across diverse patient populations and slide preparation methods.

The other 15 candidate papers cover a variety of different aspects of cancer informatics:

### Foundational Models for Cancer Informatics

- Kondepudi A, Pekmezci M, Hou X, Scotford K, Jiang C, Rao A, *et al.* Foundation models for fast, label-free detection of glioma infiltration. Nature. 2025 Jan 9;637(8045):439–45. https://doi.org/10.1038/s41586-024-08169-3 . PMID : 39537921 (8)
- Wang X, Zhao J, Marostica E, Yuan W, Jin J, Zhang J, *et al.* A pathology foundation model for cancer diagnosis and prognosis prediction. Nature. 2024 Oct 24;634(8035):970–8. https://doi.org/10.1038/s41586-024-07894-z . PMID: 39232164 (7)

### Improving Human Machine Collaboration

- Saha A, Bosma JS, Twilt JJ, Van Ginneken B, Bjartell A, Padhani AR, *et al.* Artificial intelligence and radiologists in prostate cancer detection on MRI (PI-CAI): an international, paired, non-inferiority, confirmatory study. The Lancet Oncology. 2024 Jul;25(7):879–87. https://doi.org/10.1016/S1470-2045(24)00220-1 . PMID : 38876123 (9)
- Frazer HML, Peña-Solorzano CA, Kwok CF, Elliott MS, Chen Y, Wang C, *et al.* Comparison of AI-integrated pathways with human-AI interaction in population mammographic screening for breast cancer. Nat Commun. 2024 Aug 30;15(1):7525. https://doi.org/10.1038/s41467-024-51725-8 . PMID : 39214982 (10)
- Eminaga O, Lee TJ, La V, Breil B, Xing L, Liao JC. Electronic Documentation of Intraoperative Observation of Cystoscopic Procedures Using the cMDX Information System. JCO Clin Cancer Inform. 2024 Mar;(8):e2300114. https://doi.org/10.1038/s41467-024-51725-8 . PMID : 38484216 (11)
- Gao Y, Ventura-Diaz S, Wang X, He M, Xu Z, Weir A, *et al.* An explainable longitudinal multi-modal fusion model for predicting neoadjuvant therapy response in women with breast cancer. Nat Commun. 2024 Nov 7;15(1):9613. https://doi.org/10.1038/s41467-024-53450-8 . PMID : 39511143 (12)

### Advancing Clinical Informatics Using Clinical Trials

- Djinbachian R, Haumesser C, Taghiakbari M, Pohl H, Barkun A, Sidani S, *et al.* Autonomous Artificial Intelligence vs Artificial Intelligence–Assisted Human Optical Diagnosis of Colorectal Polyps: A Randomized Controlled Trial. Gastroenterology. 2024 Jul;167(2):392-399. e2.https://doi.org/10.1053/j.gastro.2024.01.044 . PMID : 38331204 (13)
- Cui H, Zhao Y, Xiong S, Feng Y, Li P, Lv Y, *et al.* Diagnosing Solid Lesions in the Pancreas With Multimodal Artificial Intelligence: A Randomized Crossover Trial. JAMA Netw Open. 2024 Jul 19;7(7):e2422454. https://doi.org/10.1001/jamanetworkopen.2024.22454 . PMID: 39028670 (6)
- Austin JD, Finney Rutten LJ, Fischer K, Ridgeway J, Minteer S, Griffin JM, *et al.* Advancing Care Team Adoption of Electronic Health Record Systems for Cancer Symptom Management: Findings From a Hybrid Type II, Cluster-Randomized, Stepped-Wedge Trial. JCO Oncol Pract. 2025 Feb;21(2):209–17. https://doi.org/10.1200/OP.24.00280 . PMID : 39106420 (14)
- Jemaa S, Ounadjela S, Wang X, El-Galaly TC, Kostakoglu L, Knapp A, *et al.* Automated Lugano Metabolic Response Assessment in18 F-Fluorodeoxyglucose–Avid Non-Hodgkin Lymphoma With Deep Learning on 18F-Fluorodeoxyglucose–Positron Emission Tomography. JCO. 2024 Sep;42(25):2966–77. https://doi.org/10.1200/JCO.23.01978 . PMID : 38843483 (15)
- Yuan XL, Liu W, Lin YX, Deng QY, Gao YP, Wan L, *et al.* Effect of an artificial intelligence-assisted system on endoscopic diagnosis of superficial oesophageal squamous cell carcinoma and precancerous lesions: a multicentre, tandem, double-blind, randomised controlled trial. The Lancet Gastroenterology & Hepatology. 2024 Jan;9(1):34–44. https://doi.org/10.1016/S2468-1253(24)00168-7 . PMID : 37952555 (16)

### Genomic Drivers of Cancer

- Horie S, Saito Y, Kogure Y, Mizuno K, Ito Y, Tabata M, *et al.* Pan-Cancer Comparative and Integrative Analyses of Driver Alterations Using Japanese and International Genomic Databases. Cancer Discovery. 2024 May 1;14(5):786–803. https://doi.org/10.1158/2159-8290.CD-23-0902 . PMID : 38276885 (17)
- Hamilton Z, Aseem A, Chen Z, Naffakh N, Reizine NM, Weinberg F, *et al.* Comparative Analysis of Generative Pre-Trained Transformer Models in Oncogene-Driven Non–Small Cell Lung Cancer: Introducing the Generative Artificial Intelligence Performance Score. JCO Clin Cancer Inform. 2024 Dec;(8):e2400123. https://doi.org/10.1200/CCI.24.00123 . PMID : 39661913 (18)

## 4. Conclusion

These papers present promising frameworks and applications for multimodal AI, foundation models, and randomized trials of AI in cancer care. We were pleased to see an emphasis on prospective applications of AI well-represented.
